# S05-2 Tenth anniversary of ‘moment! - motor learning and mental training for people with a beginning dementia'

**DOI:** 10.1093/eurpub/ckac093.024

**Published:** 2022-08-29

**Authors:** Ute Mueller-Steck, Karen Zacharides K

**Affiliations:** Bildungsakademie des Landessportbundes Hessen e.V, Frankfurt, Germany

**Keywords:** physical activity, elderly persons, implementation, volunteers, participation

## Abstract

Currently 1.7 million people in Germany are living with dementia and two out of three such persons are nursed at home. Considering this challenging situation, we started in 2010 the project “moment! - motor learning and mental training for people with an incipient dementia”. Our aim was to develop a special qualification for people who are interested in conducting physical exercise groups for persons suffering from dementia. It has been scientifically proven that physical exercises show a positive impact on people with dementia: Training in groups is motivating and influences the emotional state in a positive way. Furthermore, social contacts are supported and maintained and prevent from social isolation. Therefore, a higher life fulfillment can be increased temporarily, and self-confidence is growing as well. Socio political goals are the integration of affected persons into existing social structures like sports associations, the raise of public awareness for dementia, and to strengthen the possibilities of integrating people suffering from dementia into regular social life. All scientific analysis proves that motion has a positive impact on the process of dementia and is a successful prophylactic and therapeutic support of the common medical treatment.

The project includes a qualification for multipliers such as care assistants, trainers in local sport associations, nursing staff and volunteers in care organisations in order to support people suffering from dementia. This training consists of five days (and a total of 40 learning units) and is offered as an attendance seminar. During the course the participants will be trained in basic knowledge and clinical pictures of dementia, above all they receive many ideas for practical motor and mental exercises. Additionally, all participants receive a workbook and a music CD for the rhythm and dance lessons. Within the last 10 years we have qualified about 440 people in 27 trainings. Many “moment!” training-groups have been built - most of them in nursing homes and a few in cooperation with a local sports club.

